# Mr. Earnest's Case of Diabetes Mellitus

**Published:** 1805-02-01

**Authors:** Robert Earnest

**Affiliations:** House Surgeon to the Sheffield General Infirmary


### *To the Editors of the Medical and Physical Journal.*

Gentlemen,

FOR the sake of our suffering fellow-creatures, nothing ought to be withheld from public notice, which may be deemed a specific for that dreadful, distressing, and lingering disease named diabetes mellitus.

If the enclosed case, (which was successfully treated with the nitrous acid) meets with your approbation, and is thought worthy a place in your very valuable Journal, by inserting it therein, you will greatly oblige,

Yours, &c.

*Dec.* 10, 1804. ROBERT EARNEST,
House Surgeon to the Sheffield General Infirmary.

---

M. D. twenty-five years of age, a female, unmarried, low in stature, and delicate in stamina. But, before I narrate the case, it will be proper here to observe, that antecedent to to her being taken ill, she was plethoric, and had a clear and florid complexion, and also enjoyed good health.

In the spring of the year 1801, she was suddenly attacked with the following symptoms, viz. pain of the head, stomach, and about the region of each kidney, great thirst, loss of appetite and strength, accompanied with much languor and depression of the spirits, a copious secretion of urine, passing off fourteen pints daily. The above symptoms continued upon her gradually, making greater ravages, and undermining her constitution, until January 2, 1804, when she was admitted into the Sheffield General Infirmary as a diabetic patient.

Her state of health at this time was very materially altered for the worse, as she was greatly emaciated; the pulse, quick, small, and hard; the skin universally yellow, hot and dry; appetite more diminished; strength also more exhausted; thirst more troublesome, particularly in the night time; œdematous swellings of the ancles and feet.

The menstrual evacuations have always been regular both as to time, quantity, and colour, until now, which are more frequent, less in quantity, thinner in consistence, and much less florid in colour.

The properties of the urine were, that it was rather limpid, sweet both to the taste and smell; and upon being evaporated by boiling, it exhibited white saccharine matter on the sides of the vessel, and the residue was like molasses.

On the second of January, the patient was put upon the following plan of cure; and the diet prescribed was principally animal food, with a generous allowance of porter. ℞. Aq. puræ lib. ii. Syr. sacchar. unc. i. Acid. nitros. dilut. dr. i. ad. iv.* M. ft. mistura, quotidie sumend.† Cap. vesp. pro re nata. Elect. e sennâ, dr. i. ad. ii.

During the administration of this remedy, the annexed table will shew the gradual return of the patient's health and strength, the state of the appetite, the quantity of acid taken, the quantity of fluids taken, and the quantity of fluid passed off from day to day.

---

* As this medicine is very apt to gripe and disturb the bowels, it is highly necessary that strict attention should be paid to its effects, as well also to the very gradual increase of the dose.

† The mixture was given as a common drink, and the patient took it at pleasure, so as to take that quantity daily.

TABLE,

### TABLE, &c.

| Date 1804 | State of the Appetite. | State of the Strength. | Quantity of Acid taken. | Quantity of Fluids taken. | | Quantity of Fluid paſſed off. | |
|---|---|---|---|---|---|---|---|
| | | | drachms | pints | oz. | pints | oz. |
| Jan. 2 | much diminiſhed | much diminiſhed | 1 | 4 | — | 14 | — |
| 3 | ditto | ditto | ditto | ditto | — | 12 | — |
| 4 | ditto | ditto | ditto | ditto | — | 5 | — |
| 5 | ditto | ditto | ditto | ditto | — | 7 | — |
| 6 | ditto | ditto | ditto | ditto | — | 4 | 8 |
| 7 | ditto | ditto | ditto | ditto | — | 5 | — |
| 8 | ditto | ditto | ditto | ditto | — | 4 | 8 |
| 9 | leſs ſo | ditto | $1\frac{1}{2}$ | ditto | — | 5 | — |
| 10 | ditto | ditto | ditto | ditto | — | 5 | — |
| 11 | ditto | ditto | ditto | ditto | — | 5 | 8 |
| 12 | ditto | ditto | ditto | ditto | — | 5 | 8 |
| 13 | ditto | ditto | ditto | ditto | — | 5 | — |
| 14 | ditto | leſs ſo | ditto | ditto | — | 5 | — |
| 15 | ditto | ditto | ditto | ditto | — | 4 | 4 |
| 16 | ditto | ditto | 2 | ditto | — | 5 | — |
| 17 | ditto | ditto | ditto | ditto | — | 5 | — |
| 18 | ditto | ditto | ditto | ditto | — | 4 | 12 |
| 19 | ditto | ditto | ditto | ditto | — | 4 | 8 |
| 20 | natural | ditto | ditto | ditto | — | 4 | 8 |
| 21 | ditto | ditto | ditto | ditto | — | 4 | 8 |
| 22 | ditto | ditto | ditto | ditto | — | 4 | 2 |
| 23 | ditto | ditto | ditto | ditto | — | 4 | — |
| 24 | ditto | ditto | ditto | ditto | — | 4 | — |
| 25 | ditto | much increaſed | $2\frac{1}{2}$ | ditto | — | 4 | — |
| 26 | ditto | ditto | ditto | ditto | — | 3 | 12 |
| 27 | ditto | ditto | ditto | ditto | — | 4 | — |
| 28 | ditto | ditto | ditto | ditto | — | 3 | 12 |
| *29 | ditto | ditto | —— | ditto | — | 3 | 12 |
| 30 | ditto | ditto | —— | ditto | — | 3 | 10 |
| 31 | ditto | ditto | —— | ditto | — | 3 | 12 |
| Feb. 1 | ditto | ditto | —— | 3 | 8 | 3 | 9 |
| 2 | ditto | ditto | —— | ditto | 8 | 3 | 12 |
| 3 | ditto | ditto | 3 | ditto | 8 | 3 | 8 |
| 4 | ditto | ditto | ditto | ditto | 8 | 3 | 12 |
| 5 | ditto | ditto | ditto | ditto | 8 | 3 | 8 |
| 6 | ditto | ditto | ditto | ditto | 8 | 3 | 8 |
| 7 | ditto | ditto | ditto | ditto | — | 3 | 8 |
| 8 | ditto | ditto | ditto | 4 | — | 3 | — |
| 9 | ditto | ditto | ditto | ditto | — | 3 | — |
| 10 | ditto | ditto | ditto | ditto | — | 3 | — |
| 11 | ditto | ditto | ditto | ditto | — | 3 | — |
| 12 | ditto | ditto | ditto | ditto | — | 3 | — |
| 13 | ditto | ditto | ditto | ditto | — | 3 | — |

* On the 29th of January the catamenia appeared, and continued five days, in proper quantities and natural in colour. During this period, the acid was omitted; and on the 3d of February, it was again repeated and continued.

The

The patient came from the country; and as she now appeared so far recovered, it was recommended to her to return home, as in all probability her native air would very much contribute to her complete recovery. Accordingly, on the fifteenth of February she went home; but she was still considered as an out-patient, and had directions given to her to persevere in the use of the acid, as the urgency of her complaint might occasionally require.

Nearly nine months having elapsed, it was thought proper to re-admit her again as an in-patient, in order to ascertain and verify the specific effects of the medicine; to this she readily assented, and on the 24th of October she appeared again at the Infirmary, when strict attention for some days to her general health in every particular was paid. The quantity of urine passed off at this time never exceeded three pints. The patient looked well, ate, drank, and slept well; and on the second of November she was discharged cured.

In three cases of excessive polydipsia, I have known the nitrous acid essentially useful.

